# Healthcare service delivery to refugee children from the Democratic Republic of Congo living in Durban, South Africa: a caregivers’ perspective

**DOI:** 10.1186/s12916-018-1153-0

**Published:** 2018-09-27

**Authors:** Anna Meyer-Weitz, Kwaku Oppong Asante, Bukenge J. Lukobeka

**Affiliations:** 1 Discipline of Psychology, University of KwaZulu-Natal, Durban, South Africa; 2 Department of Psychology, University of Ghana, P. O. Box LG 84, Legon, Accra, Ghana

**Keywords:** Refugee children, Healthcare services, Accessibility, South Africa, Democratic Republic of Congo

## Abstract

**Background:**

Refugees are generally considered a vulnerable population, with refugee children (newborn and young children) being particularly so. Access to healthcare for this population remains a challenge. The main purpose of this study was to explore refugee caregivers’ perceptions of their children’s access to quality health service delivery to their young children in Durban, South Africa.

**Methods:**

This study used an explanatory mixed methods design, purposively sampling 120 and 10 participants for the quantitative and qualitative phases, respectively. Participants were administered a self-developed questionnaire that assessed demographic information of participants, socioeconomic status and living standard, medical history of children, satisfaction and experiences with healthcare services and refugees’ networks and social support. A semi-structured interview schedule was developed to elicit in-depth and more detailed information from the participants on the quantitative areas that were investigated. Frequencies were calculated and a χ^2^ test was used to explore the factors associated with refugees’ satisfaction of the healthcare provided and thematic analysis was used to analyse the qualitative data.

**Results:**

The majority (89%) of caregivers were women, with over 70% of them aged between 30 and 35 years. Over 74% of caregivers visited public clinics for their children’s healthcare needs. The majority of caregivers (95%) were not satisfied with healthcare services delivery to their children due to the long waiting hours and the negative attitudes and discriminatory behaviours of healthcare workers, particularly in public healthcare facilities.

**Conclusion:**

These findings underscore the need to address health professionals’ attitudes when providing healthcare for refugees. Attitudinal change may improve the relationship between service providers and caregivers of refugee children in South Africa, which may improve the health-related outcomes in refugee children.

**Electronic supplementary material:**

The online version of this article (10.1186/s12916-018-1153-0) contains supplementary material, which is available to authorized users.

## Background

Globally, mass movement of people often occurs as a result of political and economic instability, poverty and armed conflict [1]. These critical conditions push people out of their home countries in search of what they believe to be a better place to live [1]. The mass relocation of people can lead to major challenges to public resources during their movement and in the various countries of destination. African countries that agree to receive refugees are faced with challenges to address the needs of their own people as well as those of refugee populations entering their countries [2].

Refugees are generally considered a vulnerable population, with refugee children (newborn and young children) being particularly so [1, 3]. Infants and young children are often the first and most frequent victims of violence, infectious diseases and malnutrition, all of which frequently accompany displaced populations and refugee movements [3, 4]. These children face far greater dangers to their safety and well-being than the average child as a result of the sudden and violent onset of emergencies and related uncertainties [3, 5]. Despite a reduction in early child mortality due to improved child survival interventions such as immunisation, nutrition control and treatment of childhood diseases [5], the improvement in child mortality remains a challenge in developing countries accounting for 41% of under-five deaths [6].

The complex interactions between refugee status and health shows that such a status may have either adverse or positive impacts on health and well-being [5, 6]. A compromised health status and access to adequate healthcare are two major areas of vulnerability that refugees face. Maintaining good health among refugees is a challenge, not only because of the health risks commonly associated with the movement of people, but also because of the economic hardships that refugees face and the undesirable conditions in which many refugees live [7]. It is therefore critical for refugee children to access the host country’s local primary healthcare services [7].

Previous studies have highlighted several factors that adversely influence access to healthcare by refugees. Lack of knowledge about refugees’ rights, low socioeconomic status, language barriers and poor understanding of a host country’s healthcare system are factors found to influence access to healthcare for migrants living in South Africa [8–10]. The attitudes of healthcare workers towards migrants and refugees can be linked to their understanding of refugee status and their accompanied legal rights, including eligibility to free, accessible and quality healthcare services as delivered to South African citizens (referred to above); their own levels of work satisfaction, where greater levels of satisfaction are expected to translate into better healthcare delivery [11]; and personal prejudice in the form of xenophobia, evident in recent expressions of violent attacks on foreign nationals, looting of businesses and homes, as well as verbal abuse, all which received wide media coverage and caused public outrage [12]. Medical xenophobia in South Africa has also been reported [10, 13, 14].

Despite the abovementioned studies, there is a dearth of research regarding refugee children’s healthcare access. The main purpose of this study is to understand caregivers’ perceptions of the healthcare needs of refugee children (10 years and younger) as well as their perceived access to primary healthcare, including their satisfaction with healthcare services for their children. The study focuses on the caregivers of refugees from the Democratic Republic of Congo (DRC) by using an explanatory mixed methods design. The findings of this study will help to formulate policies to improve service delivery to refugee children as well as to address the key health challenges faced by parents and caregivers of refugee children.

### Theoretical frameworks

The health access model and the household resources model were used as conceptual frameworks to explain the factors that affect access to healthcare for refugee children [15, 16]. The health access model by Peters et al. [15] addresses the compromised health service access of people in poor countries and contexts. It is argued that, while a lack of financial resources creates barriers to accessing healthcare, the complexities of environmental aspects in combination with individual and household characteristics denote poverty, which impacts on other factors that may inhibit access. The cycle of poverty is viewed to impact health and well-being, which in turn maintains ill health and access to healthcare. Quality of care is central to healthcare access, which in turn is determined by geographical accessibility, availability of services, financial accessibility and acceptability of services. The policy and macro environment, in combination with individual and household characteristics, determine health status but also impact healthcare access. Due to the relatively low level of socioeconomic status and available household resources [17], the health status and general well-being of migrants and refugees are compromised. In applying this model to the study, it is expected that access to healthcare will not only be influenced by the financial and geographical location but also by users’ attitudes, beliefs, expectations and characteristics of the health facilities.

The household resource model [16] explains accessibility to healthcare services in terms of material resources, investment potential and social resources. These authors argue that material resources, investment potential and social resources are important key resources that facilitate access to better healthcare. With inadequate material and investment resources, it is expected that social networks among refugees will play an important role in their access to healthcare. A strong social network may, for example, assist in the decisions to seek refuge in a particular country and obtain information, including healthcare related, social support and even employment in the new host country [18]. In this study, we expect that social networks in the form of interpersonal ties of kinship, friendship and shared community origins, which have been found to connect refugees, former refugees and non-refugees in countries of origin in the new host country [2, 19], will help in the facilitation of better healthcare for refugee children.

## Methods

### Research design and setting

This study used an explanatory mixed methods design in which the quantitative cross-sectional survey was followed by a small qualitative study. These methods were chosen because it allowed the researchers an opportunity to understand different aspects of the quantitative data in more detail [20]. As the study quantitatively investigated refugee parents’ or caregivers’ perceptions of their children’s health status, the experiences in seeking healthcare as well as accessibility and satisfaction with health service delivery are explored qualitatively. The added value of the qualitative component was to gain a deeper understanding of the caregivers’ perceptions and experiences. The study was conducted in Durban, KwaZulu-Natal province of South Africa. The province of KwaZulu-Natal has the second largest populace of the country, with 10.5 million people, approximately 19.8% of the country’s population [21], and is known to host a great number of refugees from the DRC [22, 23].

### Sampling and participants

A non-probability purposive sampling in combination with snowball sampling was used to recruit participants in this study as it allowed the researchers to select participants able to provide rich information about the phenomenon being studied [24]. This sampling strategy was used to select the research participants who are parents and/or caregivers of refugee children (aged 0 to 10 years), from the DRC, living in Durban, KwaZulu-Natal. Within the community of refugees from the DRC, the different networks that exist were used to gain access to the parents/caregivers before approaching them to seek their participation in the study. Participants were included in the study if they met the inclusion criteria, namely being a DRC refugee living in Durban, aged 18 years and over, had a child or taking care of other children, and willing to take part in the study. Based on the inclusion criteria, 120 parents or caregivers of young children (< 1 to 10 years of age) were recruited for the quantitative phase of the study. Ten of the participants from the quantitative study were purposively selected to provide further information on different aspects explored in the qualitative phase. Principles of data saturation were applied and no additional data was obtained after approximately 10 interviews [25].

### Measures

A structured questionnaire was developed by the researchers based on a good understanding of the literature, the theoretical framework, and the aims and objectives of the study. The questionnaire consisted of five main sections, namely demographic information of participants, socioeconomic status and living standard, medical history of children, satisfaction and experiences with healthcare services, and refugees’ networks and social support. The demographic information included age, sex, level of education, religious affiliation, marital status, English language proficiency and number of children had by caregivers. The socioeconomic statuses of participants focused on employment status and living standard of caregivers of refugee children. Some of the questions asked were ‘Are you currently employed?’, ‘How many people are you supporting in your household?’, ‘How many people are you sharing your accommodation with?’ The third section on medical history of children asked questions that assessed the health status of both the caregivers and the children. Some of the questions asked included ‘Has your child been immunised?’ and ‘Where was your child immunised?’ The response format for these questions was in the form of ‘Yes’ or ‘No’. Participants were also asked the type of vaccination children received and at what age this was done. Questions on accessibility, experiences and satisfaction with the healthcare services focused on caregivers’ general satisfaction with the healthcare services, healthcare consultation process, and their perceptions and experiences with private and public healthcare services. Examples of some of the questions asked included ‘On a scale of 0–10, rate your satisfaction with the healthcare service your children received from the private medical doctor, local clinic, a faith healer, local herbalist and the traditional healer’, ‘Were you able to ask all the questions you wanted to when you last visited a public clinic/hospital?’ and ‘Did the attending nurse spend enough time with you?’ The final section of the questionnaire, which focused on refugee networks and social support, elicited information about the availability of support from other refugees living in Durban. Some of the questions asked were ‘Have you received any help from your refugee community?’ and ‘How often do you meet with your family members?’ Questions about sources of information about healthcare, such as friends, family members and other individuals from church, were also asked.

For the qualitative study, a semi-structured interview schedule was developed in English, translated into French and translated back into English, based on the key research areas of the quantitative research instrument. Open-ended questions were developed in this regard to elicit in-depth and more detailed information from the participants on the quantitative areas that were investigated. Some of the questions asked were ‘What are the kinds of illnesses that your children suffer from for which you sought medical treatment?’, ‘How did the healthcare workers make you feel when you visited the clinics?’, ‘Why did you choose the private doctor?’, and ‘What were your experiences with the clinic services?’ Additional file 1 provides a full description of the questionnaire.

### Data collection and procedures

Before data collection commenced, ethical approval to conduct the study was obtained from the Ethics Committee of the University of KwaZulu-Natal, Durban, South Africa (Reference: HSS/0123/013 M). Caregivers were approached to participate by explaining the aims and objectives of the study in a language they understood, in most instances French and Swahili. Those who agreed to participate in the study were given a written informed consent to sign after being informed that their participation was voluntary and that confidentiality and anonymity would be maintained. The anonymity of the participants was guaranteed through the use of pseudonyms, and they were assured of their rights to withdraw from the study at any point in time without any negative consequences to them. Permission was also gained to audio tape the qualitative interviews. An interview was scheduled with the participants at a place and time most convenient to them. The administering of the questionnaire took an average of 35 minutes, while the qualitative interviews took approximately 45 to 60 minutes. Data collection lasted for 3 months. Qualitative data collection lasted for a further 4 weeks.

### Data analysis

The Statistics Package for Social Sciences (SPSS) version 23 was used to analyse the quantitative data. The data was first entered into Microsoft Excel before later being imported into SPSS. Frequencies and descriptive statistics were conducted to describe the sample and on all the items guided by the objectives of the study. χ^2^ tests were used to explore relationships among categorical variables, namely (1) the relationship between demographic variables (level of education, sex and age), satisfaction of healthcare provided and socioeconomic status of caregivers, (2) the relationship between demographic variables (level of education, sex and age), the different resources adopted in the research framework (i.e. Material Resources, Investment Potential and Social Resources), as well as social networks of refugee caregivers. The Mann–Whitney U test was used to test for differences between two independent groups, i.e. demographic data in relationship with satisfaction with healthcare services at both public and private facilities. It was also used to evaluate differences regarding caregivers’ experiences with the healthcare system.

All the qualitative interviews were transcribed verbatim, and thematic analysis was used to analyse the data using the guidelines of Braun and Clark [26]. The first step in analysing the data for this study involved familiarising and immersing oneself in the data to identify the common themes. In the second step, themes that shared the same words, styles and terms used by participants and the ways in which they were connected, were identified. This was followed by coding of the themes and sub-themes linked to the broad objectives of the study. The last step in the process involved the interpretation of the data and cross checking.

## Results

### Sociodemographic characteristics of participants

The demographic characteristics of the participants are presented in Table 1; 89% of the participants were females and approximately 61% of the respondents were aged 30–35 years. The majority of the participants (80.0%) were married, and 90% were actual caregivers of their own children. Approximately 71% of the respondents in the study had senior secondary education, 90% were Christians and over 70% of the participants had three children. Overall, 46.7% of the participants in the study were not able to communicate in English (i.e. could not speak, understand or write in English), while 27.5% reported than they could understand but cannot speak English, and 25.8% revealed that they could speak and write in English. The majority of the participants (86.7%) were asylum seekers (i.e. not classified as refugees by the United Nations High Commissioner for Refugees (UNHCR)), while 13.3% were officially refugees. Approximately half of the refugee caregivers decided to relocate to Durban as they already had relatives living there. A substantial group (38.5%) indicated that, because they had hentered South Africa through Mozambique, they felt safe and decided to stay in Durban.

**Table 1:** Sociodemographic information of the participants (*N* = 120)

Characteristics	Number	%
Sex		
Male	13	10.8
Female	107	89.2
Age groups		
23–29 years	21	17.5
30–35 years	73	60.8
36–40 years	14	11.7
> 40 years	12	10.0
Marital status		
Married	96	80.0
Separated	17	14.2
Divorced	6	5.0
Widower	1	0.8
Relationship with child		
Mothers	108	90.0
Fathers	12	10.0
Number of children		
3 children	85	70.8
4–5 children	28	23.3
6–7 children	7	5.9
Caregivers’ level of education		
Primary school (Grade 1–6)	5	4.2
Junior secondary school (Grade 7–9)	22	18.3
Senior certificate (Grade 12)	85	70.8
Tertiary	8	6.7
Religious affiliation		
Christian	108	90.0
Muslim	12	10.0
English language proficiency		
Write and speak English	31	25.8
Understand but cannot speak	33	27.5
Neither speak, understand nor write	56	46.7
Reasons for relocating to Durban		
Had relatives living in Durban	61	50.8
Came through Mozambique and stayed	46	38.4
Job opportunities	7	5.8
Clean city	3	2.5
Feel secure in Durban	3	2.5

### Socioeconomic and social support of participants

Information on the socioeconomic conditions, household resources and available social capital is presented in Table 2. With regards to the economic challenges refugees face, the majority of the participants (66.7%) reported that they did not have enough money for basic things like food and clothes, with only 0.8% of the respondents indicating that they had money to afford more expensive items such as a TV, radios, etc., but not enough money to buy any expensive commodities. Participants who reported having enough money for food and clothes were also more likely to report having a post-school qualification (χ^2^ = 4.406, *df* = 1; Fisher’s exact test *p* = 0.42). Interestingly, further analysis did not reveal any significant difference between those who had money for basic food and clothes and those that had enough only for the basics and their English language proficiency (χ^2^ = 1.070, *df* = 2; Fisher’s exact test *p* = 0.589). When participants were asked about their source of income, the majority of respondents (96.7%) reported that they were not fully employed. Most (85%) relied on family members/friends and 75.8% received help from their church (pastors). Additionally, 53.3% of the participants indicated that they relied on their own skills to receive an income by providing services needed by the refugee community.

**Table 2:** Frequencies of items regarding household resources and social capital

Items linked to household resources	N	Yes %	No %
Material resource items			
Working full time	120	3.3	96.7
Work part time	120	12.5	87.5
Sell some of my possessions to get money	120	5.8	94.2
Have enough money for basics like food and clothes	120	33.3	66.7
Social Resource items			
Getting financial help from family/friends in South Africa	120	85	15
Getting financial help from church/pastor	120	75.8	24.2
Getting money from family/friends in the Democratic Republic of Congo	120	0	100
Investment potential resources			
Trading	119	88.2	11.8
Have many unused skills	111	100	0.0
Using skills to provide services to refugee community	120	53.3	46.7
Social capital items (bridging)			
Know people who can help	120	97.5	2.5
Know people who are well connected with others	120	100	0.0
Know people who are willing to help when there is a need	119	72.3	27.7
Give assistance to other refugees	120	70.8	29.2
Received assistance from my community	120	64.2	35.8
Social capital items (linking)		*N*	%
Have contacted UNHCR			
Yes		9	07.5
No		111	92.5
If Yes, reason for the visit (*n* = 9)			
Social support		3	33.3
Refugee documentation		4	44.4
Information to relocate back home		2	22.2
Ever visited an office of an NGO working with refugees			
Yes		93	77.5
No		27	22.5
Received help from NGOs			
Received no assistance		48	51.6
Paid rent for few months		19	20.4
Received food vouchers when first arrived in South Africa		17	18.3
Paid school fees		9	9.7

With regards to household resources, 26%, 42.2% and 58.2% of the respondents had some material resources, social resources and investment potential, respectively. As reported in Table 2, the majority of respondents also relied on social networks for help; 97% of the participants knew someone who could help, 72.2% were aware of people willing to help whenever there is a need, and all participants were aware of people who are well connected with others. Only few (*N* = 9; 7.5%) participants had visited the UNHCR for support. The key issues that they sought help from the UNHCR for included social support (*N* = 3; 33.3%), refugee documentation (*N* = 4; 44.3%) and advice on the relocation back to their country of origin (*N* = 2; 22.2%). The results also showed that refugees received help from non-governmental organisations. However, 51.6% indicated that they never received any assistance, while those who received assistance were supported to pay rent (20.4%) and some indicated that they were given food vouchers when they first arrived in South Africa (18.3%). The remaining 9.7% reported receiving help with regards to the payment of school fees.

The qualitative findings highlighted the inconsistency in support rendered to the refugee community and the limited support they actually receive. Additionally, these refugees feel discriminated against, as shown by a narrative of a female caregiver:

“*With regards to the Refugee Social Services, I can say it does not help. This is because the services that they provide to us are based on some kind of partiality, just like the nurses do at the clinics. If you do not have a friend who is working in their offices, you will not get help. However, I have heard from some of my friends that they received help from them, where they paid 2 months’ rent for them and also gave them food*” (Female, participant 3).

### Healthcare seeking for children

The responses of caregivers regarding healthcare seeking for children are shown in Table 3. In general, the majority of the participants (74.2%) indicated that they primarily seek healthcare from the public healthcare clinics (which are generally free of charge), while the very few (2.5%) who use private medical practitioners indicated that they prefer them primarily because of the higher quality of healthcare received from them. The majority (52%) of the participants also indicated that they usually prefer seeking healthcare from private doctors who are Congolese. There were delays in seeking formal healthcare by the caregivers as 57.5% waited longer than 4 days before they sought help. Key reasons attributed to this delay were their inability to communicate in English and IsiZulu (62.3%) and the negative attitudes of healthcare workers towards refugees (30.4%). Over 65.0% used public transport as means of travel to the various healthcare centres. For participants using public transport, the average transport ranges from 10 ZAR to 20 ZAR (US$ 0.73 to US$ 1.47), considered by most as expensive when not having enough money for food.

**Table 3:** Healthcare seeking behaviours description

Healthcare seeking items	N	%
Healthcare facilities normally visited for ill health (*N* = 120)		
Public clinic	89	74.2
Public hospital	28	23.3
Private doctor	3	2.5
Actions taken after unsuccessful treatment at public facilities (*N* = 120)		
Seek help from private hospital	74	61.7
Go to another clinic	26	21.6
Get medicine from the pharmacy	9	7.5
Prayer	11	9.2
Actions taken recently when child was ill (*N* = 120)		
Went to local clinic	97	80.8
Bought medicine at the pharmacy	10	8.4
Prayed to God	9	7.5
Ask friends/neighbours for advice	4	3.3
Normal waiting time before seeking medical care (*N* = 120)		
No delay – same day	14	11.7
After 1 day	23	19.2
After 2 to 3 days	14	11.7
4 days and longer	69	57.4
Reasons for the delay of 4 days and longer (*N* = 69)		
Unable to speak English or Zulu	43	62.3
Healthcare workers hold negative attitudes towards refugees	21	30.4
Do not have valid documentation to stay in South Africa	2	2.9
Other reasons	3	4.4
Information/advice received about health issues^a^		
Friends	53	44.2
Neighbours	78	65
People at church	47	39.2
Health clinic	120	100
Private doctors	62	51.7

^a^Multiple responses to the variable, therefore total percentage is more than 100

### Satisfaction with healthcare service delivery

The caregivers were asked to rate their level of satisfaction with both public health clinics and private doctors over the last 6 months on a 10-point scale (0 = not satisfied at all, to 10 = highly satisfied). The results on the satisfaction of the healthcare service delivered to their children showed that most of the caregivers were dissatisfied with the quality of healthcare rendered to their children, particularly when referring to public healthcare services. Very low ratings were noted for the public facilities; a rating of 0 was given by 11.7%, a rating of 1 by 45% and a rating of 2 by 43.3%. However, private doctors received ratings of 5 by 3.3%, a rating of 6 by 21.7%, a rating of 7 by 34.2% and a rating of 8 by 40.8%. It is clear that the response options on the rating scale for caregivers’ satisfaction with public clinics were very restrictive and at the lower end of the rating scale, ranging from 0 to 10, showing mainly dissatisfaction with the clinic health services.

Further results showed that caregivers with a higher number of social networks were more satisfied with public healthcare delivery (*p* = 0.025) and the healthcare services by private doctors (*p* = 0.003).

Participants were asked to evaluate their level of satisfaction with the last health service consultation regarding their children at the public healthcare clinic and private doctor (Table 4). The results showed that caregivers generally had a more negative experience from their last visit to a public clinic when compared to service received from a private health facility or private general practitioner. With regards to the public clinic service, most felt that they were not able to ask the questions they wanted to (93.1%; *n* = 81), that not enough information was provided to them (91.0%; *n* = 81), that the nurses did not spend enough time with their children (100%; *n* = 89), and that their views about the healthcare needs of their children were not respected (100%; *n* = 89).

**Table 4:** Frequencies of healthcare services experiences during the last child healthcare consultation

Experiences during last clinic visit	Yes	No
	*N* (%)	*N* (%)
Public clinics (*N* = 89)		
Were you able to ask all the questions you wanted to?	6 (6.9)	81 (93.1)
Did the nurse provide you with enough information?	8 (9.0)	81 (91.0)
Did the nurse spend enough time with your child?	–	89 (100)
Will you recommend the clinic to others?	23 (25.8)	66 (74.2)
Did you have to wait too long before being attended to?	89 (100)	–
Did the nurse respect your views about your child’s healthcare needs?	–	89 (100)
Private doctor (*N* = 3)		
Were you able to ask all the questions you wanted to?	2 (66.7)	1 (33.3)
Did the doctor provide you with enough information?	2 (66.7)	1 (33.3)
Did the doctor spend enough time with your child?	3 (100)	–
Will you recommend the doctor to others?	3 (100)	–
Did you have to wait too long before being attended to?	–	3 (100)
Did the doctor respect your views about your child’s healthcare needs?	2 (100)	–

The qualitative findings support the quantitative results in the sense that dissatisfaction with the public health sector was reiterated. However, greater clarity was given to understand the issues that are problematic. The findings suggested that the participants’ dissatisfaction with healthcare services was due to a range of issues, including structural limitations as well as direct and indirect discriminatory tendencies towards refugees. With regard to structural impediments, the participants talked about the long waiting times before being attended to by healthcare workers:

“*For my first time I was with my husband, it was terrible. We stood for more than four hours since 6am to 9am. The queue was so long. After 8am they gave us numbers. We were seen by the healthcare workers after being tired, the nurse who received my child was nice, He was so cool and polite, but the one who was taking measurements was different - she was not talking to me. On the appointment day for immunisation, we face hard times at the clinic. Some nurses don’t treat foreigners like people who don’t have a country. They talk to any way - they insult people and all their healthcare communication to us was done in IsiZulu. If you ask them questions, they don’t respond to your questions, but they respond nicely when their people do same*.” (Female, participant 2)

“*I have never been happy at the local clinic as there are many things that can make you angry. You have to be there all day from 6am until the end of the day, and at the end, they will give you just Panadol (a pain killer). Sometimes you spend all your time and you worry about what the family will eat*.” (Female, participant 4)

Furthermore, the qualitative findings revealed that nurses’ negative attitudes in the public hospitals as compared to the good services provided by private medical practitioners compel them to use private hospitals rather than public.

“*There is big difference between these two clinics* [i.e. public and private]. *At the private clinic, patients feel at home and they feel more comfortable, not only because we pay the money but the way healthcare workers treat you even before you receive any medication. They welcome a patient so nicely and they take time asking you questions. Back home, in DRC, patients don’t have to wait for a long period like they do in these local clinics. When you meet with a doctor he takes time and asks you all questions and he explains to you - something which is totally different from the local clinics where they don’t give you time to ask what is wrong with you. In the private clinics, they would communicate with you in the nice manner. They show you love. They don’t have a discriminatory attitude like at the public clinic where nurses tell you “rubbish”. I am so disappointed with healthcare services at the public clinic*.” (Female, participant 8)

“*When I was at home in DRC, I was talking to my friends here (those living in South Africa) about my child who used to suffer from renal problems. They told us in South Africa, healthcare services are high* [excellent] *but when I arrived here, my main focus was about my child. But for bad luck, my child died. Prior to that I took her to the clinic with the help of one of my friends, I felt abandoned by healthcare workers. I spent more than six hours at the clinic, and no nurse cared to talk to me. It was only after my friend had complained, that they took the temperature* [of my child]*. We waited again for another two hours before we were able to see the medical doctor. The doctor gave me an appointment to see him again in four days’ time. Unfortunately, I lost my child before next appointment. Since then, I have not had any good thing to say about nurses at local clinics.*” (Female, participant 1)

Some of the participants also indicated that, notwithstanding the negative attitudes of nurses towards refugees in general, they prefer public clinics because of proximity and the fact that the service is free.

“*I chose this clinic* [public clinic] *because the services are free of charge and the clinic is closer to where we live. But I don’t like it due to many challenges we are facing at the local clinic. Nurses treat people like ‘animals’ at the clinic. If you meet the bad or not well-behaved nurse that day at the clinic, you feel like not coming back again - but there are other days when you meet with a good nurse. I cannot choose to go to the private doctor because I don’t have money, especially for children whose healthcare services are very expensive. They are so expensive at the private clinics. I have been at the private clinics myself before, so I know how expensive it is - but the services are well organised and of good quality.*” (Female, participant 5).

## Discussion

The main purpose of this study was to explore refugee caregivers’ perceptions of their children’s access to healthcare services in South Africa. Our findings showed that the majority of caregivers were not satisfied with healthcare services delivery due to the long waiting hours and the negative attitudes and discriminatory behaviours of healthcare workers, particularly in the public healthcare facilities. The discussion of these key findings is guided by the health access and the household resource models.

### Household resources and access to healthcare

The socioeconomic status among refugees is one of the key challenges they face, contributing to their health and well-being vulnerability [8, 27]. Caregivers in the study reported poor housing and living conditions, as well as a relatively low socioeconomic status. The link between poverty and ill health and mental distress is well established [28]. The socioeconomic status of refugees has been found to be one of the major barriers to accessing healthcare services and other support services in the host country [29]. The lack of financial resources is likely to impact negatively on healthcare access [15, 16].

Herein, the material resources of refugee caregivers were very limited. Material resources enable refugees to seek healthcare and pay for transport and medication. Over half of caregivers reported not having enough money for basic needs such as food or clothes, since most are unemployed, and those that do have some form of income obtain this from part-time work and trading. Anecdotal evidence suggests that many refugees work in the informal sector, with little protection, working as car guards, casual labourers in hair salons, and even resort to trading in pirated movies in attempts to keep their families alive [9, 14]. Job opportunities are restricted due to their limited proficiency in English for those living in South Africa. The lack of job opportunities for refugees should be viewed against the high unemployment rate of 25.5% in South Africa [30]. Additionally, the affirmative action legislation and widespread xenophobia among many South Africans may also impede employment opportunities of foreign nationals despite their legal status as refugees [9, 14, 31].

### Availability of healthcare services

In this study, caregivers reported having to wait for hours to access healthcare services. These views are likely to contribute to the overall dissatisfaction with the healthcare service and quality of care delivered. It has been reported that, in situations where clients have to wait longer than an hour for healthcare services, this could impact negatively on their beliefs about the quality of the service because of the emotional reactions, including stress and anger [32]. The findings that caregivers had to waste a full day waiting to be seen by a healthcare worker and being aware that they had other household responsibilities, such as preparing meals and caring for their other children, have been previously reported [33]. For full-time caregivers, it seems that spending a full day away from home results in anxiety and anger, which has been reported to impede healthcare seeking behaviour by women in particular [34]. However, waiting in long queues has been a general complaint of the South African public healthcare delivery system [35].

The language barrier due to the limited English proficiency levels among caregivers can be considered as having a negative impact on healthcare service delivery. It has been argued that language barriers contribute to the failure of treating refugees for whom English is not their first language [2]. Not only is it difficult for healthcare workers to render a good quality service if they are unable to communicate with the caregiver of about the child [10, 31, 36], it is also frustrating for caregivers not to be able to raise their concerns and ask questions. The results clearly show that caregivers were dissatisfied with the consultation process as they were not able to ask the necessary questions nor were clear guidelines and explanations given. Language differences have been reported to increase psychological distress and hinder timely healthcare seeking [37, 38].

As the ability to communicate in a shared language is linked to satisfaction with healthcare services [39], the negative views held by caregivers of healthcare delivery could partly stem from the lack of communication between client and health providers, which has previously been reported among refugees in Durban where refugees reported negative views with service delivery in public hospitals partly because of miscommunication and the absence of interpreters [2]. In the absence of professional health interpreters, family members or friends who are able to speak English are often co-opted to translate between client and healthcare worker. This process is also fraught with difficulty and misinterpretation [39–43]. A strong call has therefore been made over the years for the use of professional health interpreters in different parts of the world where there are challenges with delivering a quality service to migrant workers, asylum seekers and refugees [37, 43–45]. Public health clinics that service refugee communities should consider seeking the service of either professional interpreters or, alternatively, training members of the refugee community that have some background in health.

While not investigated herein, it is also likely that healthcare workers do experience frustration at not being able to communicate clearly with their clients. This frustration might be misinterpreted as negative, discriminatory attitudes towards refugees and even xenophobia by refugee clients. However, in the qualitative study, the view was expressed that the negative attitudes of nurses in particular is not linked to language as a barrier, but rather xenophobia directed at refugees in general. A lack of studies among health workers’ experiences in delivering healthcare to refugees in South Africa hinders a more balanced understanding of refugee healthcare delivery.

### Acceptability of healthcare services

Participants in this study indicated that the services rendered by private doctors are of a higher quality than those provided by the public healthcare system. This finding is supported by existing views of the quality of healthcare services in relation to the divide between those that can afford private healthcare and those that must seek public healthcare [46]. When considering the different levels of work satisfaction among professional nurses in the public and private sector, it has been shown that lower levels of satisfaction among nurses in the public sector negatively impact their client services, including interpersonal relationships [11].

The overall dissatisfaction with public healthcare delivery corroborates previous research findings that have reported negative attitudes of healthcare workers and discrimination against them for foreign citizens [2, 10, 14]. The results pertaining to consultation encounters at public healthcare clinics were negative as the majority of participants who asked questions related to their child’s illness was not provided with the necessary information nor felt that enough time had been spent with them during the consultation process. The language barrier in the public healthcare clinics was likely to have contributed to some of the dissatisfaction experienced by caregivers [47]. It has previously been established that refugees’ reasons for not returning to a particular clinic include long queues and long waiting times, rudeness of clinic staff and a lack of medication [48]. Therefore, caregivers’ dissatisfaction with the public health clinic services for their children seems to be aligned to issues raised by other South African clients about healthcare service delivery in general.

With regards to caregivers’ perceptions about xenophobia, it is also possible that, in a context where widespread xenophobia exists, the negative attitudes and rude behaviours of nurses could be interpreted by the caregivers as medical xenophobia [10, 14, 48]. In light of limited research insight into health providers’ views about healthcare delivery to migrants and refugees, a deeper understanding of medical xenophobia is not possible; therefore, studies among health workers are required to enhance the quality of healthcare delivery to foreign nationals in South Africa.

Our results further showed that only social networks (a category of household resource) were found to be related to caregiver’s satisfaction with child healthcare services. Specifically, caregivers with a higher number of social networks were more satisfied with public healthcare delivery and healthcare services by private doctors. It is likely that social networks help caregivers in identifying possible interpreters to assist them when seeking medical attention at the public health facilities and also assist caregivers in seeking healthcare from particular Congolese doctors, who are likely to improve the consultation experience because of more effective communication, as outlined above. The lack of diverse views about the public healthcare service could also be a consequence of the strong cohesion and seemingly closed networks among members of the DRC refugee community, further enhanced by the generalised xenophobia in South African society [9, 10]. The negative side to social capital therefore results from excessive social cohesion in groups, e.g. families, language and ethnic groups that impact various aspects of society, including economic opportunities [49], as well as ‘group think’ leading to judgement errors as trust in the group’s views inhibits independent thinking [50]. Therefore, sharing negative experiences about public healthcare services is likely to be internalised as their own negative experiences.

### Limitations of the study

While the explanatory mixed methods approach attempted to improve the quality of the findings, some limitations should be noted and care should be taken in understanding the results. The study was only conducted in one refugee community, i.e. refugees from the DRC living in Durban. The community’s experiences with child healthcare service delivery might be different for other refugee groups in Durban and those living in other parts of South Africa. Care should therefore be taken in generalising the findings to other refugee caregivers. In addition, the relatively small sample size restricts generalisation to all DRC refugee caregivers. The understanding of healthcare delivery to children from the parents’ or caregivers’ perspective provides only a one-dimensional perspective of service delivery as the view of healthcare workers is absent. Their views might have contributed to a better insight into service delivery challenges faced by healthcare workers within the constraints of current public healthcare delivery. It should be noted that some attempts were initially made to include health workers in the study, but permission to conduct such a study could not be obtained.

### Implications for policy and interventions

The findings of this study have implications for health interventions. A better understanding of organisations offering services to refugees within their locality would help them acknowledge and appreciate the work of such organisations. With regards to access to healthcare, f consideration should be first given to the use of translators/interpreters at public healthcare facilities used by refugees to improve healthcare delivery. For example, individuals from the refugee community with a background in health could be trained and employed to assist in translation and interpretation in healthcare contexts. Secondly, healthcare workers should be trained with an emphasis on patient cultural safety and prejudices as well as being made aware of discrimination in healthcare service delivery. Such training would better prepare health workers for the likely challenges they may encounter in the healthcare delivery to foreign nationals, including refugees. Thirdly, the establishment of early day care centres for financially constrained communities within urban areas, where most refugees are located, would not only enable parents and caregivers the opportunity to participate in economic activities but would also assist in the greater integration of refugees into South African society; this is likely to impact positively on the health and well-being of refugees.

## Conclusion

This study was conducted to explore refugee parents’/caregivers’ perceptions of their children’s healthcare problems and challenges regarding accessibility and quality of health service delivery in Durban, South Africa. In summary, caregivers of refugee children reported to be highly dissatisfied with the healthcare services for their children, particularly that in public healthcare facilities. Negative attitudes and discriminatory behaviours by healthcare workers were found to contribute to caregivers’ views about the poor quality of the healthcare service. This is one of the first studies to be conducted among parents/caregivers of refugees in Durban pertaining to child healthcare services, thus filling a gap in existing knowledge. These findings underscore the need to address health professionals’ attitudes when providing healthcare for refugees. Attitudinal change may improve the relationship between service providers and caregivers of refugee children in South Africa, which may improve the health-related outcomes in refugee children.

## Additional file

Additional file 1: Research Questionnaire. (DOCX 25 kb)

## Abbreviations

DRC: Democratic Republic of the Congo
NGOs: Non-governmental organizations
UNHCR: United Nations High Commissioner for Refugees
ZAR: the currency of South Africa
