# Supplementary material for: Healthcare service delivery to refugee children from the Democratic Republic of Congo living in Durban, South Africa: a caregivers’ perspective
Source: BMC Med. 2018 Sep 27;16:163. doi: 10.1186/s12916-018-1153-0 (PMC6158903; doi:10.1186/s12916-018-1153-0)
Supplement: Supplementary file 1 — Research Questionnaire. (DOCX 25 kb) [file 12916_2018_1153_MOESM1_ESM.docx]

**SECTION A: DEMOGRAPIC INFORMATION**

**Q1.Parent/Caregiver’s Age**: …………………………….

**Q2. Gender:**

Male 2. Female

**Q3.Marital Status:**

1.Single, 2. Separated, 3. Living Together; 4. Married; 5. Divorced; 6. Widowed

7. Other ……………….

**Q4.Care giver relationship with Child (ren).**

1. Mother; 2. Brother; 3. Uncle’; 4. Father; 5. Aunty; 6. Other ……………………

**How many children do you have of your own and how old are they?**

| Child | Child 1 | Child 2 | Child 3 | Child 4 | Child 5 | Child 6 | Child 7 | Child 8 |
| --- | --- | --- | --- | --- | --- | --- | --- | --- |
| Gender |  |  |  |  |  |  |  |  |
| Age |  |  |  |  |  |  |  |  |

**How many other children are responsible for and how old are they?**

| Child | Child 1 | Child 2 | Child 3 | Child 4 | Child 5 | Child 6 | Child 7 | Child 8 |
| --- | --- | --- | --- | --- | --- | --- | --- | --- |
| Gender |  |  |  |  |  |  |  |  |
| Age |  |  |  |  |  |  |  |  |

**Q5.What is your highest level of education? ……………………………**

**Q6.To which religious group do you belong**?..........................

Christian (Protestant & Catholic); Muslim; Other specify ……………………………………………

**Q7. Language: Can you understand, speak and write in English?** ……………………………………………

I can write only

Yes I can Write and Speak

I can only understand 4.

No I can't understand, speak or write in English

**Q8. SES**

I am going to read a number of statements to you. Which one best describes your household situation? (M*ark only one*)

| 1 | Not enough money for basic things like food, clothes | 1 |
| --- | --- | --- |
| 2 | Have money for food and clothes but short on many other things | 2 |
| 3 | We have the basics but not enough money for expensive items | 3 |
| 4 | Have money to save or buy expensive things | 4 |
| 5 | Other (specify) …………………………………….. | 5 |

**SECTION B. BACKGROUND**

**Q1**. Do you have any legal documentation allowing you to stay in South Africa?

Yes 2. No

**Q2**. If Yes: Please tick what you have

Permit for asylum seekers,

Refugee’s Status

ID Book (South African Green ID)

Others specify ……………………….

**Q3. Managing in South Africa**

| How do you survive in South Africa? ……………. | Yes | No |
| --- | --- | --- |
| Q3.1. Work full time in a job | 1 | 0 |
| Q3.2. Do part time work whenever I can | 1 | 0 |
| Q3.3.Get help from family/friends in South Africa | 1 | 0 |
| Q3.4. Get help from the church/pastor | 1 | 0 |
| Q3.5.Get money from family/friends in the DRC | 1 | 0 |
| Q3.6. Sell some of my things to get money | 1 | 0 |
| Q3.7. Use my skills to get some money/food | 1 | 0 |
| Q3.8. Buy and sell goods | 1 | 0 |
| Q3.9. Do you think that you have knowledge and skills that is not used in South Africa? Explain: …………………… | 1 | 0 |
| Tell me about your social networks ………. |  |  |
| Q3.10. Do you know people that can help you when you need help? | 1 | 0 |
| Q3.11. Do you know people who know others that can help you? | 1 | 0 |
| Q3.12. Are the people you know willing to help you? | 1 | 0 |

**Q4**. **If you’re not working why do you think you are not employed?**

………………………………………………………………………………………..

1=Discrimination; 2= No Job

3= I’m not able to work; 4= Many people in Durban is unemployed

5= Unable to speak English 6=Others

**Q4.1. If you’re not working: Are you looking for a job?**

1. Yes 2. No

**Q5**.**How many people are you supporting in your house?**

………………………………………

1=1-3; 2=3-6

3=More than 6;

**Q6**. With how many people are you sharing your accommodation?

……………………………………

1=1-2; 2=3-5; 3=More than 6

**Q7. How would you describe your living conditions?**

……………………………………………………………………………………………………………………………………………………………………………………………………

**SECTION C. SOCIAL NETWORKS**

**Q1. Why did you choose to come to Durban and not any other town or province in South Africa?**

………………………………………………………………………………………………...

I don’t kwow

Because of my relatives living her contacted me

Because I came for Mozambique and I didn’t know if there is others provinces in SA

Because of jobs opportunities

Other ……………………………………………..

**Q2. Did you know any person in South Africa before coming here?**

Yes; 1 No 2

**Q3. Did they know that you were coming to South Africa?**

Yes; 2=No

**Q4. Did they make you feel welcome when you arrived in South Africa?**

Yes; 2=No

**Q5. How often do you meet with them?**

1=Every Day 2=Every Sunday After church

3=Once a Month 4=If we have Ceremonies in our Community

5=we don’t meet 6=Once a Year

7=Others (Specify

**Q6. What was the biggest challenge you faced when arriving in Durban?**

………………………………………………

1.Accommodation 2.Food 3.Other ……

**Q7. What are the challenges you face now?**

**…………………………………………………**

1=House 2=Health issues

3=No Job 4=Lack of school fees for their children

5=Lack of food 6=Transport

7=Others (specify)

**Q8. Have you ever visited the United Nations high commissioner for refugees’ (UNHCR) offices**?

1=Yes 2=No

**Q9. If Yes: For what reason(s)?**

……………………………………….

1=For Social support 2=For my refugees’ documentation problems

3=Just for a visit 4=For relocation

5=for health care issues assistance 6=others (specify)

**Q10. Have you ever visited the offices of NGOs working with refugee people from other countries?**

1=Yes 2=No

**Q11. If Yes Please note the kind of assistance and Name of NGO:**

………………………………………………………………………………

**Q12. Are you belonging to any political, cultural or religious organization?**

Yes 2. No

**Q13. Have you ever receive any assistance from your community?**

Yes 2. No

**Q14. Have you ever given any assistance to any other refugee(s)?**

Yes 2. No

**Q15. If Yes: What kind of assistance did you give?**

……………………………………………………………………………….......................

**Q16. Should you ever have financial problems, who do you think will assist you?**

1. Friends from my country 2.Colleagues from work

3. Relatives or organizations 4. Church communities

5. Other: Specify, ……………………………………………

**Q17. What can be done to solve this problem of accessing health care services by refugees?**

To engage interpreters in public hospitals to overcome the language problems

To engage or abroad Congolese nurse or other Francophone nurses from others countries in the public hospitals

To awareness among South African nurses to respect medical ethics and respect for refugees rights

I don’t know, (Nothing)

To use all those mechanism: 1,2 &3

Others (specify)

**SECTION D. MEDICAL HISTORY QUESTIONNAIRE**

Q1. **When you arrived in South Africa did you and your family received a complete health assessment?**

| Medical Assessment | Yes | No |
| --- | --- | --- |
| Q1.1. Physical medical check-up? | 1 | 2 |
| Q1.2. Mental health assessment? | 1 | 2 |

**Q2. Have your children been immunised?**

1.Yes 2. No

**Q3. Place of immunisation**:

1. In South Africa 2. In DRC, 3. Other ………………….

Q4. If Yes: For what have they been immunized? (Ask to see Immunisation Card or any documentation)

| Disease | Yes | No |
| --- | --- | --- |
| BCG | 1 | 2 |
| Polio | 1 | 2 |
| Measles | 1 | 2 |
| OPV | 1 | 2 |
| All | 1 | 2 |
| Other ………………………. | 1 | 2 |

**Q5. What is your understanding of immunisation?**

| I don’t know/ I forget | 1 |
| --- | --- |
| Immunization protects children against diseases | 2 |
| To cure diseases | 3 |
| To improve children’s health |  |

**Q5.1. Have all your children completed their immunisation?**

1. Yes; 2. No

**Q6. Have any one of your children been ill lately?**

1.Yes 2. No

**Q6.1. If Yes: What did you do?**

Asked my friend/neighbour for help

I went to the local clinic

I prayed to God

I bought medicine from the Pharmacy

**Q7. Once your child is ill, for how long do you normally wait before taking him/her to the clinic or doctor?**

**……………………………………………….**

**Q7.1. If longer than 4 days: What are the reasons for waiting this long?**

**…………………………………………………………………………………………..**

1. Because of the Distance to the clinic 2. Because I don’t have transport money

3. Because they can’t help my child 4. Because they don’t like refugees in the clinic

5. Because I don’t have valid documents 6. Others

**Q8. Where do you normally go when your child needs medical care?**

1. Public clinic 2. Hospital

3. Private Doctor 4. Local herbalist

5. Local traditional healer

**Q8.1. If Public Clinic: Why did you choose to go to the clinic?**

……………………………………………………………………………

1. Because the services are free 2. Because of the good quality of services

3. Because I like it 4. Because is not far from my place

5. Because I don’t have money to go to the private doctor

**Q8.3. If Private Doctor: Where is the doctor from?**

**………………………………………………………………………………….**

| Congolese | 1 |
| --- | --- |
| From another African country? | 2 |
| SA - Indian? | 3 |
| SA - White? | 4 |

**Q8.4 If you visited a private doctor – What were your reasons for doing so?**

**…………………………………………………………………………………………**

1. Because the services are free 2. Because of the good quality of services

3. Because is close to my home.

**Q9. Where did you learn about the services offered by the clinic?**

**…………………………………………………………………………………….**

1. Because I have been there once 2. From my community

3. From my friends 4.From my church

5. In the street

**Q10. How did you get to the clinic?**

1. By taxi 2. Own car

3. Walking 4. Other specify

**Q10.1. If used transport: How much did the transport cost you to go to the clinic?**

1. R 10 2. R 20

3. < R20 4. Other

**Q11. Share some of your experiences at the clinic.**

…………………………………………………………………………………………..

**Q12. If you were unhappy why?**

**……………………………………………………………………………………….**

1= I don’t know 2= To spend all day there without getting any help

3= For Being victim of insult at the clinic as a refugee

4= Others (specify) ………………………………………………………

**Q13. Did your child get better?**

1=Yes; 2=No

**If No: What did you do? ……………………………………………**

1=Nothing (just stay with him at home) 2=I prayed to God at home

3=I changed clinic 4=Bought medicine from the pharmacy

5=I went to the traditional doctors 6=I went to the private doctor/hospital

7=Others (specify)

**Q14. Do you feel that you can be open and honest at the local clinic**?

1.Yes 2. No

**Q15. Where do you get information from about health issues?**

|  | Yes | No |
| --- | --- | --- |
| From my friends | 1 | 0 |
| From the nurse at the clinic | 1 | 0 |
| From the private doctor | 1 | 0 |
| From my neighbours | 1 | 0 |
| From the people at church | 1 | 0 |
| Media | 1 | 0 |

**Q16. Have you ever lost a child?**

1.Yes; 2. No

**Q16.1. If Yes what was the cause of death?**

Illness

Accident

Other Specify:

**SECTION E: SATISFACTION WITH HEALTH CARE SERVICES**

When you think back over the last 6 months: How would you rate your satisfaction with the health services you child/children younger 10 years and younger received? Use a scale from 0 to 10 (0 not satisfied at all and 10 highly satisfied) to indicate your satisfaction.

**1. The private doctor(s): (Not applicable – did not seek the help of a private doctor)**

| 0 | 1 | 2 | 3 | 4 | 5 | 6 | 7 | 8 | 9 | 10 |
| --- | --- | --- | --- | --- | --- | --- | --- | --- | --- | --- |

**2. The local clinic: (Not applicable – did not seek the help of the local clinic)**

| 0 | 1 | 2 | 3 | 4 | 5 | 6 | 7 | 8 | 9 | 10 |
| --- | --- | --- | --- | --- | --- | --- | --- | --- | --- | --- |

**3. A faith healer: (Not applicable – did not seek the help of a faith healer)**

| 0 | 1 | 2 | 3 | 4 | 5 | 6 | 7 | 8 | 9 | 10 |
| --- | --- | --- | --- | --- | --- | --- | --- | --- | --- | --- |

**4. The local herbalist (Not applicable – did not seek the help of a herbalist)**

| 0 | 1 | 2 | 3 | 4 | 5 | 6 | 7 | 8 | 9 | 10 |
| --- | --- | --- | --- | --- | --- | --- | --- | --- | --- | --- |

**5. The traditional healer (Not applicable – did not seek the help of a traditional healer)**

| 0 | 1 | 2 | 3 | 4 | 5 | 6 | 7 | 8 | 9 | 10 |
| --- | --- | --- | --- | --- | --- | --- | --- | --- | --- | --- |

**6. Satisfaction with clinic and private doctor**

| 1. **Local clinic** |  |  |
| --- | --- | --- |
| The last time your children/child was taken to the clinic ……. | Yes | No |
| 1.Were you able to ask all the questions you wanted to ask? | 1 | 0 |
| 2.Did the nurse provide you with enough information? | 1 | 0 |
| 3.Do you feel that the nurse spent enough time with you? | 1 | 0 |
| 4.Will you recommend the services of this clinic to others?  What would you say? | 1 | 0 |
| 5.Did you feel that you had to wait too long before being helped?  Why do you say this? ………………………… | 1 | 0 |
| 6.Did you feel that the nurse respected your opinions about your child’s health care needs? Why:…………………………………………………………………. | 1 | 0 |
| **B. Private Doctor** |  |  |
| The last time your children/child was taken to the private doctor … | Yes | No |
| 1.Were you able to ask all the questions you wanted to ask? | 1 | 0 |
| 2.Did the doctor provide you with enough information? | 1 | 0 |
| 3.Did you feel that the doctor spend enough time with you? | 1 | 0 |
| 4. Will you recommend the services of this doctor to others?  What would you say: …………………………………………………………… | 1 | 0 |
| 5.Did you feel that you had to wait too long before being helped?  Why do you say this? ……………………………………………………. |  |  |
| 6.Did you feel that the doctor respected your opinions about your child’s health care needs? ; Why: ……………………………………………………………………… | 1 | 0 |
| 7. How would you compare the Health care you received in your country to the health care in South Africa? ………………………………….……………………....................... | | |
